# Childhood adversity contributes more strongly than cognition to transdiagnostic mental health symptoms in young adults

**DOI:** 10.21203/rs.3.rs-11207066/v1

**Published:** 2026-10-06

**Authors:** Mica Komarnyckyj, Marta Litwinczuk, Ryan Shepherd, Tobias Banaschewski, Arun L.W. Bokde, Rüdiger Brühl, Sylvane Desrivières, Herta Flor, Penny Gowland, Antoine Grigis, Andreas Heinz, Frauke Nees, Dimitri Papadopoulos Orfanos, Luise Poustka, Michael N. Smolka, Nathalie Holz, Nilakshi Vaidya, Henrik Walter, Robert Whelan, Paul Wirsching, Gunter Schumann, Rebecca Elliott

**Affiliations:** University of Manchester; University of Manchester; University of Manchester; Heidelberg University; Trinity College Dublin; Physikalisch-Technische Bundesanstalt; King's College London; Heidelberg University; University of Nottingham; Université Paris-Saclay; University of Tübingen; Ludwig-Maximilians-Universität (LMU) in Munich; Université Paris-Saclay; University Hospital Heidelberg; Technische Universität Dresden; Heidelberg University; Charité Universitätsmedizin Berlin; Charité – Universitätsmedizin Berlin, corporate member of Freie Universität Berlin, Humboldt-Universität zu Berlin, Berlin Institute of Health; Trinity College Dublin; Charité – Universitätsmedizin Berlin, corporate member of Freie Universität Berlin, Humboldt-Universität zu Berlin, Berlin Institute of Health; Charité Universitätsmedizin Berlin; University of Manchester

## Abstract

Childhood adversity is associated with a broad range of mental health problems and may contribute to transdiagnostic risk via effects on cognitive development. However, it remains unclear how adversity and cognition jointly contribute to symptoms. Using the IMAGEN community sample (n = 827, mean age = 18.5), we examined cross-sectional associations between adversity, cognition and current symptoms in young adults. Three partial least squares regression models were compared: Adversity-only (threat and deprivation derived from Childhood Trauma Questionnaire); Cognition-only (CANTAB^®^ performance); and combined Adversity-and-Cognition. When modelling symptoms across nine disorders, adversity emerged as the dominant predictor; the Adversity-and-Cognition model provided only modest improvement over Adversity-only for within-sample (R^2^ = 0.189 vs 0.181) and out-of-sample predictive performance (R^2^ = 0.056 vs 0.054). Adversity-and-Cognition showed a disorder-specific predictive value for depression, conduct disorder and hyperactivity/inattention (ADHD symptoms). However, for social phobia, specific phobia, agoraphobia, OCD and PTSD, incorporating cognition did not improve predictive performance beyond adversity alone.

## Introduction

Mental health problems are often experienced with overlapping and co-occurring symptoms that do not fit neatly into single diagnostic categories. Transdiagnostic approaches, cutting across traditional diagnostic boundaries, have gained prominence as a means of understanding shared processes underlying psychopathology and developing interventions which can be applied across multiple disorders^1–3^.

Childhood adversity (including trauma, abuse, maltreatment, household dysfunction and neglect) is increasingly viewed as a transdiagnostic risk for mental health problems, based on its broad association with a range of disorders and their comorbidity^4–6^. One influential model proposes that disruption to social, emotional and cognitive development during periods of childhood adversity, confers this transdiagnostic risk for later life psychopathology^7,8^. Additionally, broad cognitive dysfunction is a standalone transdiagnostic phenomenon, emerging consistently across disorders and cognitive domains^9^. However, it remains unclear how adversity and cognition jointly contribute to heterogeneous mental health outcomes.

To better characterise the developmental effects of childhood adversity, it can be conceptualised across the broad dimensions of *threat* and *deprivation*^7,8^. *Threat* involves witnessing or directly experiencing violence or abuse. Emotional development may be disrupted by threatening events, resulting in altered threat sensitivity, hypervigilance and difficulties interpreting and regulating emotions later in life^7,10^. *Deprivation* is the absence of vital caregiver support or critical resources (e.g., food, clean clothing, warm housing) due to neglect, caregiver-child separation or caregiver psychopathology. The lack of formative social and cognitive inputs during periods of deprivation is proposed to affect the development of core cognitive skills and executive functions^8,10–12^.

There is growing evidence that threat and deprivation are associated with distinct patterns of psychopathology and brain function^13–16^. However, despite broad dimensional patterns emerging, mental health outcomes following childhood adversity are highly heterogeneous; some people develop later life difficulties whilst others do not. Research suggests that vulnerability to psychopathology following adversity is shaped by interacting cognitive, genetic, social and environmental factors^8,17–19^. Of these factors, cognition is of particular interest because it holds potential to offer an objective, low-cost and scalable way to capture clinically relevant variation in symptoms.

Emerging longitudinal evidence suggests that baseline cognition can predict mental health outcomes following adversity. In maltreatment-exposed children, atypical social cognition and reward processing can predict a worsening in internalising and externalising symptoms 1.5 years later^18^. Furthermore, cognitive performance measured 1 month after a traumatic event improves the prediction of posttraumatic stress disorder (PTSD) diagnosis 14 months after, beyond classification based solely on demographic and clinical variables^20^. These studies provide important evidence that cognition holds value when predicting the future onset or worsening of mental health symptoms following adversity. However, within the current research landscape, it remains unclear whether measures of current cognitive function add unique clinically relevant information about current mental health symptoms beyond adversity history alone.

Addressing this cross-sectional question is important because baseline cognitive data is not always available for clinicians or researchers. In contrast, current assessments of cognitive performance following adversity are more practical to obtain and may reflect combined influence of experiences, pre-existing vulnerabilities, compensatory processes and symptom burden. Answering this question could clarify the value of including current cognitive measures alongside adversity history in clinical research. It could also help elucidate whether specific cognitive domains are more relevant to specific disorders and explain the heterogeneity in mental health outcomes following childhood adversity.

To address this gap, we investigate three research questions using the IMAGEN community sample of European young adults^21^: (i) whether adversity (threat and deprivation) and cognitive function jointly contribute to variation in mental health symptoms, (ii) whether combining adversity and cognitive measures improves cross-sectional symptom prediction beyond adversity measures alone, (iii) whether the contribution of cognition differs when characterising individual disorder symptoms, compared with symptoms across multiple disorders in a transdiagnostic model.

We treat current cognitive performance as a clinically relevant marker of risk and illness burden, as we cannot fully disentangle pre-existing vulnerabilities from effects of current symptoms on test performance. We use partial least squares (PLS) regression, which can identify latent components that capture covariation between multiple outcomes and predictors, making it well-suited for modelling transdiagnostic symptom patterns that co-occur across mental health conditions.

## Methods

### Participants

This study used data from IMAGEN, a community-based project investigating psychological, biological and environmental influences on development and mental health^21^. Approximately 2000 adolescent participants were recruited at baseline and gave written consent at six European study sites (Berlin, Dresden, Dublin, London, Mannheim, Paris). Data were collected over 10 years in four waves, with target participant ages of 14 (baseline), 16 (follow-up 1), 19 (follow-up 2) and 23 (follow-up 3).

As this study is focussed on childhood trauma and adversity, analyses used data from follow-up 2, when participants completed the self-report retrospective Childhood Trauma Questionnaire (CTQ). The total sample size at follow-up 2 was n = 1,525. Participants were included in the present analyses if they had complete data for the relevant: mental health outcomes within the Strengths and Difficulties Questionnaire (SDQ) and Development and Well-Being Assessment (DAWBA) (reduced n = 1366); CTQ variables (reduced n = 1167); and Cambridge Neuropsychological Test Automated Battery (CANTAB) task variables (final sample = 827). Missingness in the CANTAB variables was the main contributor to the reduced analytic sample. Notably, participants had to visit the university sites to complete CANTAB tasks during supervised assessment which may have been a barrier to completion, whereas the clinical questionnaires were completed remotely online.

### Mental health measures

DAWBA measured internalising symptoms including depression, anxiety, specific phobia, social phobia, agoraphobia, PTSD and obsessive-compulsive disorder (OCD). The DAWBA algorithm estimated the likelihood of DSM-IV and ICD-10 diagnoses using self-report information only at IMAGEN follow-up 2. The algorithm assigned one of six probability bands, ranging from < 0.1% (band 0) to > 70% (band 5) chance of having the disorder^22^. Externalising symptoms were measured using a self-report, adult version of the SDQ with continuous scores (0–10) for conduct disorder and hyperactivity/inattention (higher scores indicate greater symptom severity)^23^.

### Childhood trauma and adversity measures

CTQ quantifies exposure to trauma before age 19 using five subscales (emotional abuse, physical abuse, sexual abuse, emotional neglect and physical neglect), with total scores ranging from 5 (no exposure) to 25 (severe exposure)^24^. Building on methods used in prior adversity research, here *threat* was calculated as the sum of CTQ abuse subscales (emotional, physical and sexual abuse), with scores ranging from 15 (no abuse) to 75 (extreme abuse). *Deprivation* was calculated as the sum of CTQ neglect subscales (emotional and physical neglect), with scores ranging from 10 (no neglect) to 50 (extreme neglect)^15,16,25^. Importantly, model generalisability was improved when using the dimensions of threat and deprivation, rather than the five CTQ subscales (Supplementary Table 1), therefore, all subsequent analyses used the dimensional adversity measures.

### Cognitive measures

CANTAB measures of cognitive function used in this study are given below and were summarised from details previously published^26,27^.

**Affective Go/No-Go (AGN)** task measures inhibitory control and attentional biases to emotional words. Participants respond to target emotional categories (Go trials) and withhold responses to non-targets (No-Go trials). *AGN Positive Latency* and *AGN Negative Latency* reflect mean response times to positive and negative Go stimuli, respectively, with longer latencies indicating slower processing speed.

**Cambridge Gambling Task (CGT)** measures decision-making and risk-taking. On each trial, participants guess the location of a hidden token and bet a proportion of their points on that decision (starting with 100), gaining or losing points based on accuracy. Higher scores for *CGT Quality* and *CGT Risk Adjustment* indicate improved decision-making. Higher scores on other measures indicate more impulsive betting (*CGT Delay Aversion)*, increased risk taking *(CGT Risk Taking)*, higher average betting ratio (*CGT Proportion Bet)* and slower decision-making (*CGT Deliberation Time)*^28^.

**Rapid Visual Information Processing (RVP)** task measures sustained attention. Participants are shown digits in a pseudo-random order and must respond as quickly as possible when they detect a target sequence of three consecutive digits. *RVP A’* (i.e., A-prime) is a signal-detection index of sensitivity to target sequences, with values closer to 1 indicating better discrimination^29^.

**Spatial Working Memory (SWM)** task assesses the ability to retain and manipulate visuospatial data. Participants search for a yellow token hidden within a set of coloured boxes, with difficulty increasing as the number of boxes increases. Poorer performance is indicated by higher scores for *SWM Strategy* (i.e., less systematic/efficient search strategies) and *SWM Errors* (i.e., revisiting boxes where the contents are known).

### Statistical Analyses

PLS regression is a multivariate method that identifies latent components by finding linear combinations of predictor variables that are maximally correlated with linear combinations of response variables. Then, the PLS regression method uses these latent components to predict the response variables. Here, PLS regression was used to predict mental health outcomes from models containing either: 1) threat and deprivation (Adversity-only model); 2) CANTAB cognitive measures (Cognition-only model); or 3) a combination of the two (Adversity-and-Cognition model) (Fig. 1). Age and sex were included as predictors in all models.

First, nested cross-validation was performed to select the number of PLS components and validate this learning method. Before model fitting in both outer and inner loops, site effects were regressed out from predictors and outcomes using dummy-coded recruitment centres, with regression parameters estimated on the training data and then applied to the corresponding validation/test data. All variables were then normalised (z-scored) using training-sample means and standard deviations.

The outer validation loop employed 100 repeats of 5-fold cross-validation, splitting the whole sample into training and test samples. The training sample was used for fitting the models, and the test sample was used to assess out-of-sample predictive performance, which was estimated using explained variance (coefficient of determination, R^2^) and accuracy of the validation sample’s predicted scores (Pearson’s R between true and predicted scores). The inner cross-validation loop was used for hyperparameter tuning (number of PLS components) with 10 repeats of 3-fold cross-validation, using the training sample. Models were fitted using a range of 1–4 components, with the optimal number of components determined based on the highest explained variance within the inner test samples across 10 repeats.

Final models were fitted to the whole sample (827 participants) following site regression and normalisation. The optimal number of components was determined as the average number of components used across all outer cross-validation repetitions. Final predictive performance was estimated using explained variance and accuracy of whole sample predictions for each outcome and these estimates were averaged to obtain overall mental health prediction performance estimates. The relationship between predictors and mental health outcomes was assessed by examining the component loadings from this final model. Additionally, variable importance projection (VIP) score was estimated to quantify each predictor’s contribution to the model. Predictors with VIP ≥ 1.0 are considered important for the overall model across all components.

Permutation testing was conducted to determine whether the model's out-of-sample predictions performed significantly above chance. One thousand repeats of nested cross-validation were performed. During each repeat, the outcome variables were shuffled before model training and out-of-sample prediction. The explained variance of these predictions was estimated for each repeat, which generated a null distribution of explained variance. This null distribution of explained variance was compared against the empirical distribution to estimate its probability (p-value), which was calculated as the proportion of null model repeats that achieved a performance equal to or better than the empirical models.

To test whether the Adversity-and-Cognition model produced greater out-of-sample predictive performance than the Adversity-only model, we used a permutation-based approach rather than pairwise comparison, because repeated cross-validation does not generate independent samples which can inflate significance of the t-test^30,31^. First, we calculated the difference in out-of-sample explained variance (empirical ΔR^2^) between the Adversity-and-Cognition and Adversity-only models, where the same folds were used for both models in each repeat, and averaged the empirical ΔR^2^ across 100 cross-validation repeats. Then, a null distribution of ΔR^2^ was generated by randomly permuting the rows of the cognitive predictors in Adversity-and-Cognition model 5,000 times, while outcomes and all other predictors (demographics and adversity scores) remained intact. This abolished any relationship between outcomes and cognitive predictors while preserving the relationship with remaining predictors. For each permutation, both the Adversity-only and cognition-permuted Adversity-and-Cognition models were fitted using the same cross-validation split. Finally, for each permutation we calculated the difference in R^2^ between the cognition-permuted Adversity-and-Cognition model and the refitted Adversity-only model, which resulted in the null distribution of ΔR^2^. Following recommended best practices, during permutation, model training was performed with nested inner cross-validation in the same manner as the empirical model^32^. Finally, the average empirical ΔR^2^ was compared with the null distribution of ΔR^2^, by calculating the proportion of ΔR^2^ values in the null distribution that were equal to or greater than the average empirical ΔR^2^.

### Note

Benjamini–Hochberg false discovery rate (FDR) correction was applied separately to each family of statistical tests. FDR-adjusted *p*-values are reported as *q*-values, with *q* < .05 considered statistically significant.

## Results

### Sample characteristics

827 participants (54.29% females) met the inclusion criteria with a mean age of 18.53 years (SD = 0.74, range = 18–23). All participants were of European descent with recruitment per site as follows: 9.92% Berlin, 20.68% Dresden, 10.88% Dublin, 17.78% London, 19.83% Mannheim, 20.92% Paris.

### Mental health outcomes

In this community sample of young adults there was a low prevalence of mental health symptoms, indicated by low mean scores across all outcomes (Table 1) and the distribution of scores across DAWBA and SDQ bands (see Supplementary Table 2 and Table 3, respectively).

Females reported increased symptoms of depression, anxiety, social phobia, specific phobia, agoraphobia and OCD (all *q* < .001). No significant sex differences were observed for PTSD, conduct problems or hyperactivity/inattention (Table 1).

### Childhood trauma and adversity dimensions

This sample experienced relatively low adversity exposure indicated by low mean scores in CTQ subscales, threat and deprivation dimensions (Table 2). For the CTQ subscales, emotional neglect (M = 7.88) and emotional abuse (M = 7.01) were most commonly reported, while sexual abuse was the least frequent (M = 5.28) (Table 2). Females reported significantly higher levels of sexual abuse compared to males (*q* = .005); whereas males reported significantly more emotional neglect (*q* = .018). No significant sex differences were observed for other subscales (Table 2). For the adversity dimensions, females reported significantly higher threat compared to males (*q* = .018); no significant sex difference was observed for deprivation (Table 2). Within this sample, threat and deprivation were moderately positively correlated, *r*(825) = .53, 95% CI [.48, .58], *p* < .001.

### Cognitive measures

For a summary of cognitive performance and correlations between cognitive variables, see Supplementary Table 4 and Supplementary Fig. 1, respectively.

### Whole sample predictive performance

For combined mental health outcomes across all nine disorders (representing transdiagnostic risk), the Adversity-and-Cognition model explained 18.90% of variance in outcomes, achieved moderate accuracy (R = 0.41) and was fitted with four components. The Adversity-only model showed similar performance, explaining 18.06% of the variance with comparable accuracy (R = 0.40). The Cognition-only model explained less variance (15.61%) and achieved lower accuracy (R = 0.36). The Adversity-only and Cognition-only models were both fitted with three components. Whole-sample predictive performance is summarised in Supplementary Table 5, and beta values used to make model predictions are reported in Supplementary Tables 6–8.

For all individual mental health outcomes, the Adversity-and-Cognition model also explained the most variance (Supplementary Table 9). The comparisons for whole sample predictive performance in this section are descriptive; formal statistical comparisons of model performance are reported below based on out-of-sample predictions.

### Out-of-sample predictive performance: transdiagnostic models

For combined mental health outcomes, the Adversity-and-Cognition model achieved the highest out-of-sample predictive performance (mean R^2^ = .056 ± 0.004, mean *r*_*s*_ = .211 ± .005), followed by the Adversity-only model (mean R^2^ = .054 ± 0.004, mean *r*_*s*_ = .203 ± .003) and the Cognition-only model (mean R^2^ = .029 ± 0.004, mean *r*_*s*_ = .177 ± .005). All out-of-sample models performed significantly better than chance (all *q* < .05, Supplementary Table 10).

To test whether cognitive predictors added predictive value beyond adversity predictors alone, we compared out-of-sample R^2^ between the Adversity-and-Cognition and Adversity-only models across the same 100 cross-validation repeats. The mean improvement after adding cognition was small (mean ΔR^2^ = .003 ± 0.003; 95% percentile interval: −0.004 to 0.008) but significant (null mean ΔR^2^ = − .007 ± 0.003; one-sided permutation *p* = .0004).

### Out-of-sample predictive performance: individual outcome models

All out-of-sample models for individual mental health outcomes performed significantly better than chance (all *q* < .05, Supplementary Table 11). The Adversity-and-Cognition model achieved greater out-of-sample predictive performance compared with the Adversity-only model for: depression (mean ΔR^2^ = .011 ± 0.003; 95% interval = 0.003–0.016; *p* = .003, *q* = .010), conduct disorder (mean ΔR^2^ = .016 ± 0.004; 95% interval = 0.006–0.024; *p* = .002, *q* = .010) and hyperactivity/inattention (mean ΔR^2^ = .019 ± 0.004; 95% interval = 0.010–0.026; *p* < .001, *q* = .003). Smaller improvements in predictive performance were found for anxiety (mean ΔR^2^ = .005 ± 0.004; 95% percentile interval = −0.004–0.013; *p* = .023, *q* = 0.051). However, including cognition with adversity predictors did not improve predictive performance for specific phobia, social phobia, agoraphobia, OCD or PTSD (Fig. 2 and Table 3).

### Adversity-and-Cognition model structure

This section focuses on the Adversity-and-Cognition model, which was the winning model based on model comparison (see Supplementary Fig. 2 and Fig. 3 for Adversity-only and Cognition-only model loadings).

The transdiagnostic mental health signal was carried substantially by demographics and retrospective self-report adversity, with the four highest variables in terms of VIP being sex (VIP = 1.41), threat (VIP = 1.36), deprivation (VIP = 1.26) and age (VIP = 1.03). VIP scores for all cognitive predictors were below 1.0 meaning their contribution for predicting outcomes was smaller compared with other variables. VIP was, however, normalised so that the mean squared VIP across all predictors equals 1, therefore VIP values below 1.0 do not imply absolute unimportance.

Across all components, higher threat and deprivation exposure was associated with greater mental health symptom severity (Fig. 3).

Component 1 explained 66.92% of variance in predictors and 13.32% of variance in mental health outcomes and reflected a broad transdiagnostic liability for psychopathology. This component was characterised by largest predictor loadings from older age, increased adversity and cognitive loadings which reflected a speed-accuracy trade-off: slower responding (AGN Positive Latency, AGN Negative Latency), combined with improved decision-making (CGT Quality, CGT Risk Adjustment). Poorer SWM strategy also loaded on this component, suggesting an additional contribution of less efficient spatial working memory. Anxiety, conduct disorder and hyperactivity/inattention had the strongest outcome loadings.

Component 2 explained 6.73% of variance in predictors and 3.95% of variance in outcomes. Sex was the strongest predictor loading; given coding of female = 0 and male = 1, the direction of predictor outcome loadings indicate stronger associations for females, with the strongest associations observed for internalising symptoms. This component was characterised by poorer working memory and adaptive decision-making, greater risk-taking behaviour *(CGT Risk Taking and CGT Proportion Bet*) and slower responding *(AGN Positive Latency and AGN Negative Latency)*.

Component 3 and Component 4 together accounted for only 1.62% of the variance in outcomes and therefore their loadings are not interpreted here.

## Discussion

In a community sample of European young adults, we investigated whether there is a benefit of characterising current mental health symptoms based on both childhood adversity and current cognitive function. Adversity emerged as the dominant transdiagnostic predictor of mental health, whereas cognition provided only modest value in cross-sectional symptom prediction. For both within-sample and out-of-sample predictions of combined symptoms across nine disorders, a model incorporating both adversity and cognitive predictors provided only incremental improvements over using adversity predictors on their own. The contribution of cognition, however, varied across individual disorders, improving prediction for depression and externalising symptoms but not for agoraphobia, PTSD, OCD, social phobia or specific phobia. These findings suggest that childhood adversity captures a large portion of the transdiagnostic signal associated with mental health symptoms, while current cognitive function may provide disorder-specific information.

Childhood adversity was a robust predictor of mental health symptoms in young adulthood, with higher threat and deprivation associated with more severe symptoms. Within the Adversity-and-Cognition model, threat and deprivation emerged as important predictors, whereas cognitive variables showed lower relative importance. Our findings show that adversity is associated with multiple symptom domains simultaneously and contributes to shared variance across disorders, supporting prior literature positioning adversity as a risk factor for many psychopathological disorders^4–7^. These results highlight the importance of investigating how symptoms co-occur following the exposure to adversity.

Threat and deprivation are theorised to differentially influence child cognitive development, with some evidence of distinct mental health trajectories^7,13,14^. This study, however, did not identify any components with distinct adversity dimensions. Instead, threat and deprivation shared similar loadings across components, suggesting that both contributed to similar symptom patterns and the model captured the effects of a high general adversity load rather than a dimensionally specific variance in symptoms. The PLS model used was designed to predict symptoms across nine disorders using multiple adversity and cognitive predictors, rather than focusing on specific mechanisms e.g., altered emotional/fear processing (hypothesised link to threat) or executive functioning (hypothesised link to deprivation). Given the high co-occurrence between threat and deprivation experiences, and the fact that both dimensions are broadly associated with internalising and externalising symptoms^11^, this broad transdiagnostic approach may have been less suited to detecting distinct threat- and deprivation-specific pathways.

Despite the dominant contribution of adversity, cognition showed a shared influence on symptom patterns. The combined model demonstrated that cognition provided a modest amount of complementary information when compared to the Adversity-only model. In component 1, representing a broad vulnerability for psychopathology, more severe symptoms for all mental health outcomes were associated with higher levels of adversity and slower emotional processing and poorer spatial working memory but also improved performance in sustained attention and decision-making. This pattern may reflect a speed-accuracy trade-off, whereby slower responding is accompanied by more accurate or cautious performance^33^, rather than the broad cognitive dysfunction often reported across mental health disorders^9,34–36^. When interpreting these findings, it is important to consider that current cognitive functioning is likely influenced by several factors which cannot be disentangled within this cross-sectional design. For example, genetics and early environmental factors (e.g., education, socio-economic status (SES), caregiver support and adversity)^8,37^; state-level daily fluctuations (e.g., due to sleep, well-being and stressors); and cognitive deficits arising due to psychological and physical health conditions^38^.

We found that including cognition benefitted modelling of some disorders but not others. There are several potential explanations. Firstly, some disorders may be more related to adversity-related experiences, rather than altered cognitive processes. Secondly, the cognitive measures used here may not cover enough trauma-related and affective cognitive processes (e.g., emotion/fear processing and threat detection) to benefit modelling across the full range of disorders. It is also important to highlight that poorer performance of the combined Adversity-and-Cognition model for some disorders may reflect the addition of weakly informative predictors, which can reduce out-of-sample generalisability, rather than indicating disorder-specific differences.

Cognition appeared to provide additional information beyond adversity for symptoms of depression, hyperactivity/inattention (i.e., a proxy for ADHD) and conduct problems. For these disorders, measures of executive functioning appeared to capture clinically relevant variance in current symptoms which was not fully explained by adversity alone. This suggests that the brief CANTAB measures used here may improve characterisation of current symptom profiles in subclinical populations when used alongside self-report measures of adversity. This finding is consistent with previous evidence of altered cognitive functioning in clinical populations (including depression, anxiety and ADHD), also demonstrated using CANTAB tasks, such as Spatial Working Memory and Rapid Visual Information Processing^29,34,35,39^.

In contrast, Adversity-only models performed as well as the combined Adversity-and-Cognition models in predicting symptoms of specific phobia, social phobia (i.e., social anxiety), agoraphobia, OCD and PTSD. This may indicate that these symptom profiles are more strongly aligned with adversity-related experiences than effects of adversity on cognition. This finding was unsurprising given that onset and symptom exacerbation of several of these disorders is influenced by trauma, adversity and difficult interpersonal experiences^40–43^. However, it remains possible that the most relevant cognitive processes for these affective or trauma-related disorders have not been captured by the available CANTAB measures, which were predominantly emotionally neutral, focussing on general executive functioning and attentional processes. Cognitive tasks targeting socio-emotional and trauma-specific mechanisms, such as emotional regulation, vigilance to threat, learning and social cognition, are likely to hold greater predictive utility; however, these measures were not available within the IMAGEN dataset.

The present study was designed to characterise cross-sectional associations between adversity, cognition and mental health symptoms, rather than evaluate prospective prediction. Longitudinal prediction is now needed to explore whether this approach holds potential for understanding early symptom expression and risk profiles before more severe symptoms develop. Missingness of socioeconomic status (SES) data (57%) prevented its inclusion despite its known influence on cognition, therefore future studies should incorporate SES and more ethnically diverse samples (IMAGEN is exclusively participants of European descent). Despite low levels of adversity and mental health symptoms within the sample, robust out-of-sample prediction was achieved, suggesting multivariate PLS models can characterise subclinical symptom profiles cross-sectionally. The emergence of these associations within this community sample - with low variance and floor effect context - suggests robustness of the predictive model which is not dependent on extreme adversity scores or clinical severity. Future work should evaluate whether these associations generalise to clinical populations, where higher levels of adversity and symptom severity may result in different patterns of association and alter the predictive value of cognitive variables.

In conclusion, childhood adversity (threat and deprivation) emerged as the key transdiagnostic predictor of mental health symptoms in young adults. However, the contribution of current cognitive functioning was disorder specific. Combining Adversity-and-Cognition showed a disorder-specific cross-sectional predictive value for depression, conduct disorder and hyperactivity/inattention (i.e., ADHD symptoms). In contrast, Adversity-only models performed as well as Adversity-and-Cognition models for agoraphobia, PTSD, OCD, social phobia and specific phobia. These findings highlight the need for mechanistically informed models that integrate adversity history with a variety of cognitive measures (including emotional, trauma-related and executive function) to explain individual differences in mental health outcomes.

## Supplementary Material

Supplementary Files

This is a list of supplementary files associated with this preprint. Click to download.

- AdversityCognitionIMAGENsupplementary.docx

## Figures and Tables

**Figure 1:**
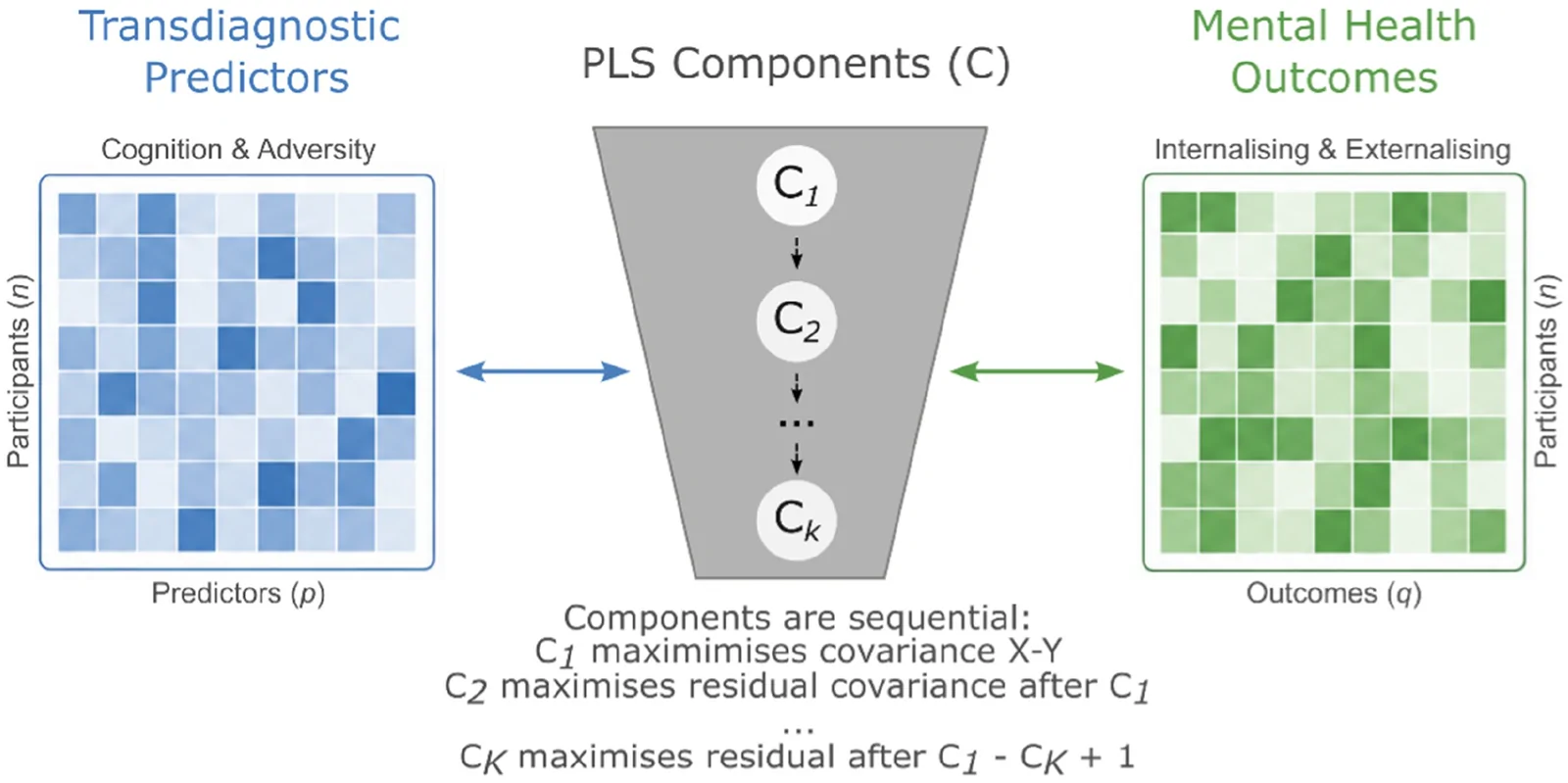
Methods: Schematic of partial least squares regression *Note*. Schematic overview of multivariate partial least squares (PLS) regression. Latent components were sequentially extracted to maximise covariance between predictor variables and outcome measures.

**Figure 2:**
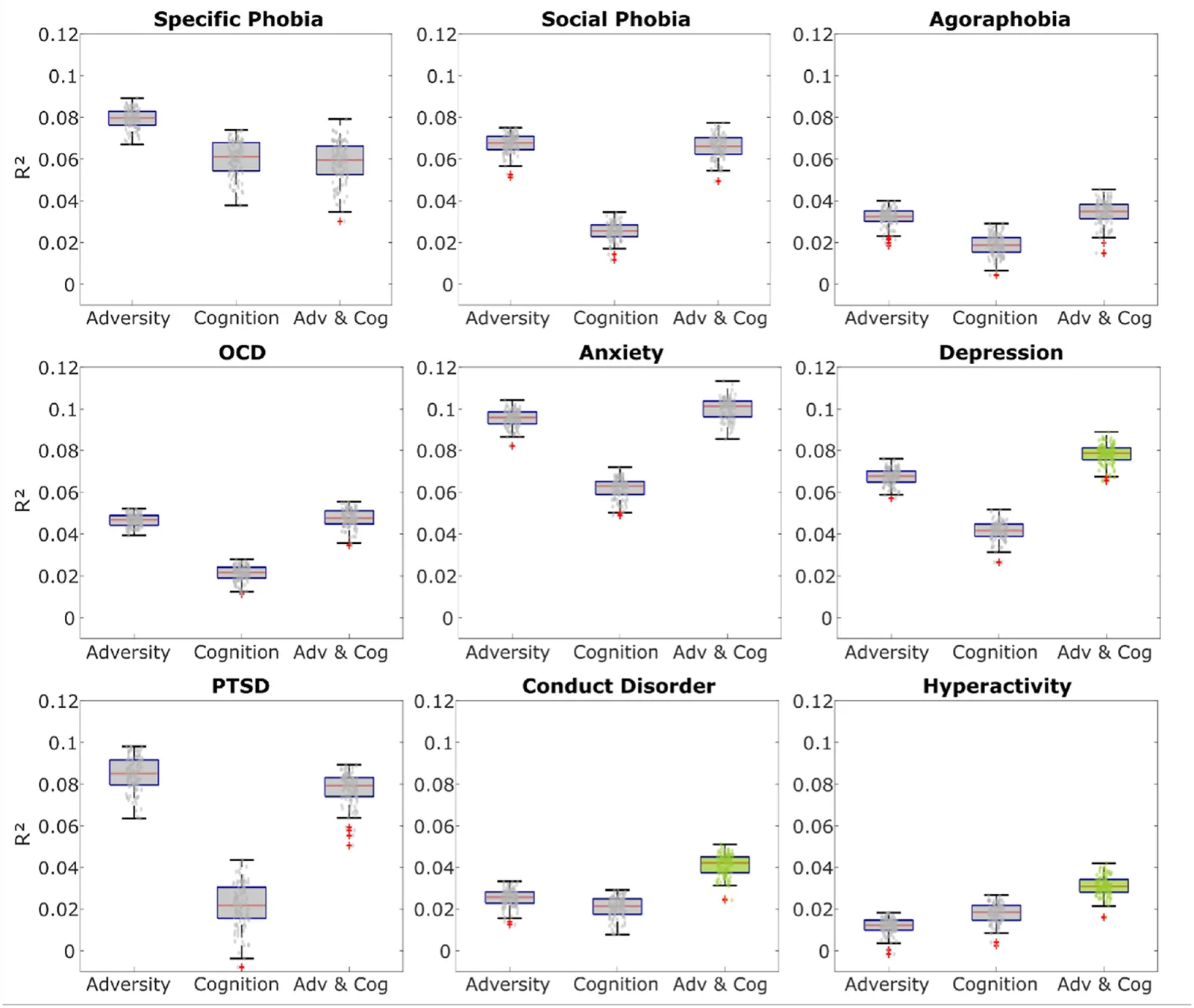
Model comparisons: Out-of-sample predictive performance across individual mental health outcomes. *Note*. Whisker plots show the distribution of out-of-sample explained variance (R^2^) across 100 cross-validation repeats. Green plots indicate outcomes for which the Adversity-and-Cognition model showed significantly greater predictive performance than the Adversity-only model based on permutation testing. Adv & Cog = model including both adversity and cognitive predictors; OCD = obsessive-compulsive disorder; PTSD = post-traumatic stress disorder.

**Figure 3:**
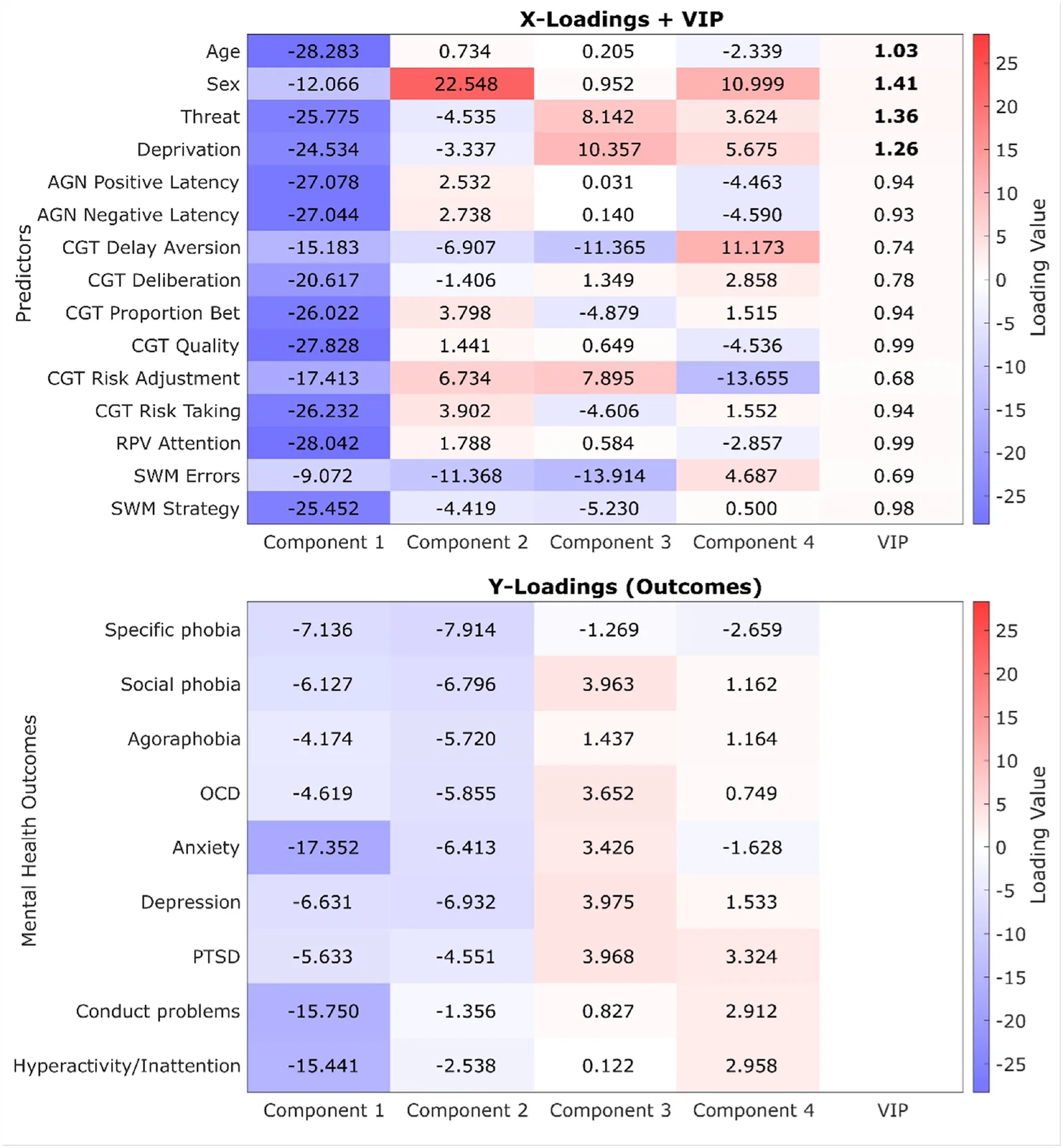
PLS regression model loadings: prediction of mental health outcomes with adversity and cognition. *Note*. Components are ordered by the proportion of variance explained in mental health outcomes. Top matrix: X-loadings for demographic, adversity and cognitive predictors. Bottom matrix: Y-loadings for mental health outcomes. Colours indicate the direction and magnitude of loadings, with warmer colours representing positive values and cooler colours representing negative values. Numerical values correspond to X- and Y-loading coefficients. Within PLS components, when X- and Y-loadings have the same sign (both positive or both negative) it indicates a positive association between those predictors and outcomes. If the X-loading and Y-loadings have opposite signs, it indicates a negative association.

**Table 1:** Mental health symptom mean scores (whole sample and split by sex)

	All	Male M (S.D)	Female M (SD)	Male vs Female	
	M (SD)	M (SD)	M (SD)	*p*	*q*
**Development and Well-Being Assessment** ^1^					
Depression	0.68 (1.09)	0.46 (0.89)	0.86 (1.20)	< .001	< .001
Anxiety	1.36 (0.69)	1.17 (0.48)	1.52 (0.79)	< .001	< .001
Social phobia	0.39 (0.83)	0.23 (0.58)	0.52 (0.96)	< .001	< .001
Specific phobia	0.40 (0.64)	0.19 (0.41)	0.57 (0.74)	< .001	< .001
Agoraphobia	0.10 (0.31)	0.05 (0.22)	0.13 (0.36)	< .001	< .001
OCD	0.20 (0.52)	0.11 (0.38)	0.27 (0.61)	< .001	< .001
PTSD	0.12 (0.43)	0.09 (0.33)	0.15 (0.49)	.049	.063
**Strengths and Difficulties Questionnaire** ^2^					
Conduct problems	1.69 (1.21)	1.73 (1.26)	1.66 (1.18)	.421	.421
Hyperactivity/inattention	3.37 (2.12)	3.28 (2.08)	3.44 (2.15)	.275	.310

Note. Differences between males and females were tested using Welch’s two-sample t-tests. P values were adjusted across the nine comparisons using the Benjamini–Hochberg false discovery rate procedure and are reported as q values. OCD = obsessive compulsive disorder. PTSD = post-traumatic stress disorder.

1 The Development and Well-Being Assessment assigns individuals a probability band indicating the likelihood of having each disorder: 0 '<0.1%' 1 '~0.5%' 2 '~3%' 3 '~15%' 4 '~50%' 5 '70%+'.

2 Strengths and Difficulties Questionnaire measures have a score of 0–10 and higher scores indicate greater severity.

**Table 2:** Childhood Trauma Questionnaire and adversity mean scores (whole sample and split by sex)

	All	Male	Female	Male vs Female	
	M (SD)	M (SD)	M (SD)	*p*	q
Age	18.53 (0.74)	18.57 (0.77)	18.49 (0.71)	.088	.117
**Childhood Trauma Questionnaire Subscales** ^1^					
Emotional abuse	7.01 (2.65)	6.83 (2.40)	7.16 (2.83)	.064	.104
Physical abuse	5.33 (1.05)	5.31 (0.90)	5.35 (1.16)	.530	.606
Sexual abuse	5.28 (1.57)	5.08 (1.01)	5.44 (1.91)	< .001	.005
Emotional neglect	7.88 (2.98)	8.20 (3.06)	7.62 (2.90)	.006	.018
Physical neglect	5.96 (1.73)	5.98 (1.59)	5.94 (1.84)	.758	.758
**Adversity Dimensions** ^2^					
Threat	17.62 (4.04)	17.22 (3.17)	17.96 (4.63)	.007	.018
Deprivation	13.84 (4.12)	14.17 (4.13)	13.56 (4.08)	.033	.067

Note. Differences between males and females were tested using Welch’s two-sample t-tests. P values were adjusted across the eight comparisons using the Benjamini–Hochberg false discovery rate procedure and are reported as q values.

1 For childhood trauma questionnaires subscales, scores range from 5 (no exposure) to 25 (severe exposure).

2 Adversity dimensions are composite scores of childhood trauma questionnaires subscales (threat = emotional abuse + physical abuse + sexual abuse, deprivation = emotional neglect + physical neglect).

**Table 3:** Added predictive value of cognition beyond adversity for individual mental health outcomes: permutation-test results

Mental Health Outcome	Observed ΔR^2^, M ± SD	Null ΔR^2^, M ± SD	p	q
Specific Phobia	− .021 ± 0.010	− .014 ± 0.009	.805	.805
Social Phobia	− .002 ± 0.005	− .005 ± 0.005	.275	.309
Agoraphobia	.002 ± 0.004	−.004 ± 0.005	.084	.152
OCD	.001 ± 0.004	−.004 ± 0.005	.117	.175
Anxiety	.005 ± 0.004	−.007 ± 0.006	.023	.051
Depression	.011 ± 0.003	−.005 ± 0.005	.003	**.010**
PTSD	− .007 ± 0.009	−.015 ± 0.011	.243	.309
Conduct Disorder	.016 ± 0.004	−.005 ± 0.006	.002	**.010**
Hyperactivity/Inattention	.019 ± 0.004	−.003 ± 0.005	< .001	**.004**

Note. ΔR^2^ = R^2^ for the Adversity-and-Cognition model minus R^2^ for the Adversity-only model. Observed values are based on 100 cross-validation repeats; null values are based on 5,000 permutations. *p* values are one-tailed tests of whether cognition improves predictive performance. *q* values are Benjamini–Hochberg FDR-adjusted *p* values across nine outcomes. Bold indicates *q* < .05.

## Data Availability

The data used in this study were obtained from the IMAGEN Consortium. The data are not publicly available due to participant privacy and data protection restrictions but can be accessed by researchers upon application and approval of a formal project proposal by the IMAGEN Executive Committee. Detailed instructions for data access and application forms are available on the IMAGEN Project Website.

## Code availability

The scripts used for data analysis in this study are available at [URL address].

## References

[1] Caspi A. The p Factor: One General Psychopathology Factor in the Structure of Psychiatric Disorders? Clin Psychol Sci 2, 119–137 (2014). 10.1177/2167702613497473 PMC4209412 25360393

[2] Insel T. Research domain criteria (RDoC): toward a new classification framework for research on mental disorders. Am J Psychiatry 167, 748–751 (2010). 10.1176/appi.ajp.2010.09091379 20595427

[3] Dalgleish T., Black M., Johnston D. & Bevan A. Transdiagnostic approaches to mental health problems: Current status and future directions. J Consult Clin Psychol 88, 179–195 (2020). 10.1037/ccp0000482 PMC7027356 32068421

[4] van Nierop M. Childhood trauma is associated with a specific admixture of affective, anxiety, and psychosis symptoms cutting across traditional diagnostic boundaries. Psychol Med 45, 1277–1288 (2015). 10.1017/s0033291714002372 25273550

[5] Bendall S. Childhood trauma is prevalent and associated with co-occurring depression, anxiety, mania and psychosis in young people attending Australian youth mental health services. Aust N Z J Psychiatry 57, 1518–1526 (2023). 10.1177/00048674231177223 37243364

[6] Stangeland H. Prevalence and risk of psychiatric disorders in young people: prospective cohort study exploring the role of childhood trauma (the HUNT study). The British Journal of Psychiatry 225, 476–483 (2024). 10.1192/bjp.2024.98 PMC11663475 39114956

[7] McLaughlin K. A., Colich N. L., Rodman A. M. & Weissman D. G. Mechanisms linking childhood trauma exposure and psychopathology: a transdiagnostic model of risk and resilience. BMC Med 18, 96 (2020). 10.1186/s12916-020-01561-6 PMC7110745 32238167

[8] McLaughlin K. A., Sheridan M. A. & Lambert H. K. Childhood adversity and neural development: deprivation and threat as distinct dimensions of early experience. Neurosci Biobehav Rev 47, 578–591 (2014). 10.1016/j.neubiorev.2014.10.012 PMC4308474 25454359

[9] Abramovitch A., Short T. & Schweiger A. The C Factor: Cognitive dysfunction as a transdiagnostic dimension in psychopathology. Clinical Psychology Review 86, 102007 (2021). 10.1016/j.cpr.2021.102007 33864968

[10] Duffy K. A., McLaughlin K. A. & Green P. A. Early life adversity and health-risk behaviors: proposed psychological and neural mechanisms. Ann N Y Acad Sci 1428, 151–169 (2018). 10.1111/nyas.13928 PMC6158062 30011075

[11] Lee A. H. Do dimensions of childhood adversity differ in their direct associations with youth psychopathology? A meta-analysis. Dev Psychopathol 37, 871–901 (2025). 10.1017/s0954579424000737 38584264

[12] Duprey E. B., Handley E. D., Russotti J., Manly J. T. & Cicchetti D. A Longitudinal Examination of Child Maltreatment Dimensions, Emotion Regulation, and Comorbid Psychopathology. Res Child Adolesc Psychopathol 51, 71–85 (2023). 10.1007/s10802-022-00913-5 PMC9464798 35278164

[13] Uddin H. Associations of threat and deprivation-related childhood exposures with children’s mental health and flourishing: The moderating role of family resilience. Children and Youth Services Review 166, 107912 (2024). 10.1016/j.childyouth.2024.107912

[14] Schafer J. L. Threat and deprivation are associated with distinct aspects of cognition, emotional processing, and psychopathology in children and adolescents. Dev Sci 26, e13267 (2023). 10.1111/desc.13267 PMC10028496 35417607

[15] Banihashemi L. Childhood Threat Is Associated With Lower Resting-State Connectivity Within a Central Visceral Network. Frontiers in Psychology Volume 13 (2022). 10.3389/fpsyg.2022.805049 PMC8927539 35310241

[16] Banihashemi L. Opposing relationships of childhood threat and deprivation with stria terminalis white matter. Hum Brain Mapp 42, 2445–2460 (2021). 10.1002/hbm.25378 PMC8090789 33739544

[17] Hofgaard L. S., Nes R. B. & Roysamb E. Introducing two types of psychological resilience with partly unique genetic and environmental sources. Sci Rep 11, 8624 (2021). 10.1038/s41598-021-87581-5 PMC8060303 33883571

[18] Gerin M. I., Viding E., Armbruster-Genc D. J. N., Roiser J. P. & McCrory E. J. Advancing prevention science for maltreatment exposed children: predicting mental health symptoms with a combined neurocognitive vulnerability index. Eur Child Adolesc Psychiatry (2025). 10.1007/s00787-025-02838-y PMC13212408 41123603

[19] Meehan A. J. Developing an individualized risk calculator for psychopathology among young people victimized during childhood: A population-representative cohort study. J Affect Disord 262, 90–98 (2020). 10.1016/j.jad.2019.10.034 PMC6916410 31715391

[20] Schultebraucks K. Assessment of early neurocognitive functioning increases the accuracy of predicting chronic PTSD risk. Mol Psychiatry 27, 2247–2254 (2022). 10.1038/s41380-022-01445-6 PMC11129320 35082440

[21] Schumann G. The IMAGEN study: reinforcement-related behaviour in normal brain function and psychopathology. Molecular Psychiatry 15, 1128–1139 (2010). 10.1038/mp.2010.4 21102431

[22] Goodman A., Heiervang E., Collishaw S. & Goodman R. The 'DAWBA bands' as an orderedcategorical measure of child mental health: description and validation in British and Norwegian samples. Soc Psychiatry Psychiatr Epidemiol 46, 521–532 (2011). 10.1007/s00127-010-0219-x 20376427

[23] Goodman R., Meltzer H. & Bailey V. The Strengths and Difficulties Questionnaire: a pilot study on the validity of the self-report version. Eur Child Adolesc Psychiatry 7, 125–130 (1998). 10.1007/s007870050057 9826298

[24] Bernstein D. P., Fink L., Handelsman L. & Foote J. Childhood Trauma Questionnaire (CTQ). APA PsycTests. (1994). 10.1037/t02080-000

[25] Lambert H. K., King K. M., Monahan K. C. & McLaughlin K. A. Differential associations of threat and deprivation with emotion regulation and cognitive control in adolescence. Dev Psychopathol 29, 929–940 (2017). 10.1017/S0954579416000584 PMC5243929 27424571

[26] Cambridge Cognition. CANTAB^®^, %3Chttps://cambridgecognition.com/digital-cognitive-assessments/%3E (2025).

[27] Sharma A. in Encyclopedia of Autism Spectrum Disorders (ed Volkmar Fred R.) 498–515 (Springer New York, 2013).

[28] Romeu R. J., Haines N., Ahn W. Y., Busemeyer J. R. & Vassileva J. A computational model of the Cambridge gambling task with applications to substance use disorders. Drug Alcohol Depend 206, 107711 (2020). 10.1016/j.drugalcdep.2019.107711 PMC6980771 31735532

[29] Gau S. S. F. & Huang W. L. Rapid visual information processing as a cognitive endophenotype of attention deficit hyperactivity disorder. Psychological Medicine 44, 435–446 (2014). 10.1017/S0033291713000640 23561037

[30] Dietterich T. G. Approximate Statistical Tests for Comparing Supervised Classification Learning Algorithms. Neural Computation 10, 1895–1923 (1998). 10.1162/089976698300017197 9744903

[31] Nadeau C. & Bengio Y. Inference for the Generalization Error. Machine Learning 52, 239–281 (2003). 10.1023/A:1024068626366

[32] Ojala M. & Garriga G. C. Permutation Tests for Studying Classifier Performance. Journal of Machine Learning Research 11, 1833–1863 (2010).

[33] Wenzlaff H., Bauer M., Maess B. & Heekeren H. R. Neural characterization of the speed-accuracy tradeoff in a perceptual decision-making task. J Neurosci 31, 1254–1266 (2011). 10.1523/JNEUROSCI.4000-10.2011 PMC6623618 21273410

[34] Arnone D. Assessment of cognitive domains in major depressive disorders using the Cambridge Neuropsychological Test Automated Battery (CANTAB): Systematic review and meta-analysis of cross-sectional and longitudinal studies. Prog Neuropsychopharmacol Biol Psychiatry 138, 111301 (2025). 10.1016/j.pnpbp.2025.111301 40010427

[35] Fried R., Hirshfeld-Becker D., Petty C., Batchelder H. & Biederman J. How Informative Is the CANTAB to Assess Executive Functioning in Children With ADHD? A Controlled Study. J Atten Disord 19, 468–475 (2015). 10.1177/1087054712457038 22923781

[36] Effah R., Ioannidis K., Grant J. E. & Chamberlain S. R. Exploring decision-making performance in young adults with mental health disorders: a comparative study using the Cambridge gambling task. Psychol Med 54, 1890–1896 (2024). 10.1017/S0033291724000746 PMC11413343 38721767

[37] Wang Y., Anney R. & Pat N. Relationship between cognitive abilities and mental health as represented by cognitive abilities at the neural and genetic levels of analysis. eLife 14, RP105537 (2025). 10.7554/eLife.105537 PMC12618009 41236810

[38] Schretlen D. J., Finley J. A., Del Bene V. A. & Varvaris M. The Ubiquity of Cognitive Impairment in Human Illness: a Systematic Review of Meta-Analyses. Arch Clin Neuropsychol 40, 863–877 (2025). 10.1093/arclin/acae113 39667720

[39] Luo W. Disorder-specific impaired neurocognitive function in major depression and generalized anxiety disorder. J Affect Disord 318, 123–129 (2022). 10.1016/j.jad.2022.08.129 36057290

[40] American Psychiatric Association. Diagnostic and statistical manual of mental disorders: DSM-5^™^, 5th ed., (American Psychiatric Publishing, Inc, 2013).

[41] Zenoni M., Lopez M. R., Archer S. & Milton A. L. Trauma-related pathways in obsessive-compulsive disorder: A systematic review of aetiology, symptom dimensions and severity. Compr Psychiatry 146, 152664 (2026). 10.1016/j.comppsych.2026.152664 41570718

[42] Keyes C., Nolte L. & Williams T. I. The battle of living with obsessive compulsive disorder: a qualitative study of young people's experiences. Child Adolesc Ment Health 23, 177–184 (2018). 10.1111/camh.12216 32677307

[43] Zhang H. The relationship between childhood trauma and social anxiety in college students: the mediating role of evaluation fear. BMC Psychiatry 25:280 (2025). 10.1186/s12888-025-06668-9 PMC11938748 40133865

